# Assessment of intratumoral heterogeneity with mutations and gene expression profiles

**DOI:** 10.1371/journal.pone.0219682

**Published:** 2019-07-16

**Authors:** Ji-Yong Sung, Hyun-Tae Shin, Kyung-Ah Sohn, Soo-Yong Shin, Woong-Yang Park, Je-Gun Joung

**Affiliations:** 1 Samsung Genome Institute, Samsung Medical Center, Seoul, Korea; 2 Department of Health Science and Technology, Samsung Advanced Institute of Health Science and Technology, Sungkyunkwan University, Seoul, Korea; 3 Department of Software and Computer Engineering, Ajou University, Suwon, Korea; 4 Department of Digital Health, Samsung Advanced Institute of Health Science and Technology, Sungkyunkwan University, Seoul, Korea; 5 Big Data Research Center, Samsung Medical Center, Seoul, Korea; 6 Department of Molecular Cell Biology, Sungkyunkwan University School of Medicine, Seoul, Korea; Sechenov First Medical University, RUSSIAN FEDERATION

## Abstract

Intratumoral heterogeneity (ITH) refers to the presence of distinct tumor cell populations. It provides vital information for the clinical prognosis, drug responsiveness, and personalized treatment of cancer patients. As genomic ITH in various cancers affects the expression patterns of genes, the expression profile could be utilized for determining ITH level. Herein, we present a novel approach to directly detect high ITH defined as a larger number of subclones from the gene expression pattern through machine learning approaches. We examined associations between gene expression profile and ITH of 12 cancer types from The Cancer Genome Atlas (TCGA) database. Using stomach adenocarcinoma (STAD) showing high association, we evaluated the performance of our method in predicting ITH by employing three machine learning algorithms using gene expression profile data. We classified tumors into high and low heterogeneity groups using the learning model through the selection of LASSO feature. The result showed that support vector machines (SVMs) outperformed other algorithms (AUC = 0.84 in SVMs and 0.82 in Naïve Bayes) and we were able to improve predictive power by using both combined data from mutation and expression. Furthermore, we evaluated the prediction ability of each model using simulation data generated by mixing cell lines of the Cancer Cell Line Encyclopedia (CCLE), and obtained consistent results with using real dataset. Our approach could be utilized for discriminating tumors with heterogeneous cell populations to characterize ITH.

## Introduction

Intratumoral heterogeneity is defined as different tumor cells as being capable of exhibiting distinct morphological and phenotypic profiles, including cellular morphology, gene expression, metabolism, proliferation, and metastatic potential [1]. The research on tumor heterogeneity is essential to figure out the composition of the tumor for personalized treatment of cancer patients. The ITH studies are attracting attention as an important concept in the understanding of drug resistance and cancer recurrence, which is a critical issue in cancer treatment, and in deciphering the mechanism of acquiring resistance to target chemotherapy [2]. Therefore, understanding the heterogeneity of tumors is very important, not only in designing the therapeutic approach to find the potential target for chemotherapy, but also in the field of clinical diagnosis for prediction of prognosis and therapeutic response of cancer patients [3].

Stomach adenocarcinoma (STAD) is the most common cancer in the world and has a very high mortality rate [4]. It is particularly interesting to understand the molecular characteristics of STAD from a genomic point of view through the application of next generation sequencing (NGS) technology and to study the causes and consequences of stomach adenocarcinoma through the assessment of ITH. In STAD, as well as in several other cancer types, ITH study is invaluable for identifying suitable biomarkers and correct therapeutic strategy because each subpopulation of cells (i.e., a subclone of cancer cells) contains distinct genetic information [5]. Currently, researchers have calculated the purity/ploidy of tumors [6] to know how many tumor cells are inherently contained on the basis of the copy number alteration data and somatic mutation data obtained from the carcinoma. In addition, the degree of tumor heterogeneity has been determined by estimating the number of sub-clones existing within a tumor [7].

Nonetheless, the measurement of ITH by identifying sub-clones using genome profiles requires complex and intensive analytical procedures, such as the calculation of tumor purity, ploidy, variant allele frequency (VAF), cancer cell fraction (CCF), and clustering. Recent study has suggested that high level of ITH affects the recurrence risk based on the gene expression profile [8]. Another approach has demonstrated that the degree of ITH could be measured by the biological network entropy using gene expression data [9]. Thus, their previous studies suggest that not only genomic data but also gene expression patterns can be used as invaluable information to identify ITH.

A method is needed for easily determining the tumor heterogeneity by utilizing various types of omics data. We propose a method to classify tumors with high heterogeneity exhibited as a larger number of subclones according to the mutation and expression profiles of genes that may be associated with stomach adenocarcinoma. We tried to distinguish samples with high tumor heterogeneity by combining the mutation data set and the gene expression data set to obtain meaningful insights for determining tumor cell diversity. During this process, our method could easily find potential biomarkers through a feature selection and conveniently determine candidate tumor samples with high heterogeneity.

## Results

### Relationship between ITH and RNA expression

**Fig 1:**
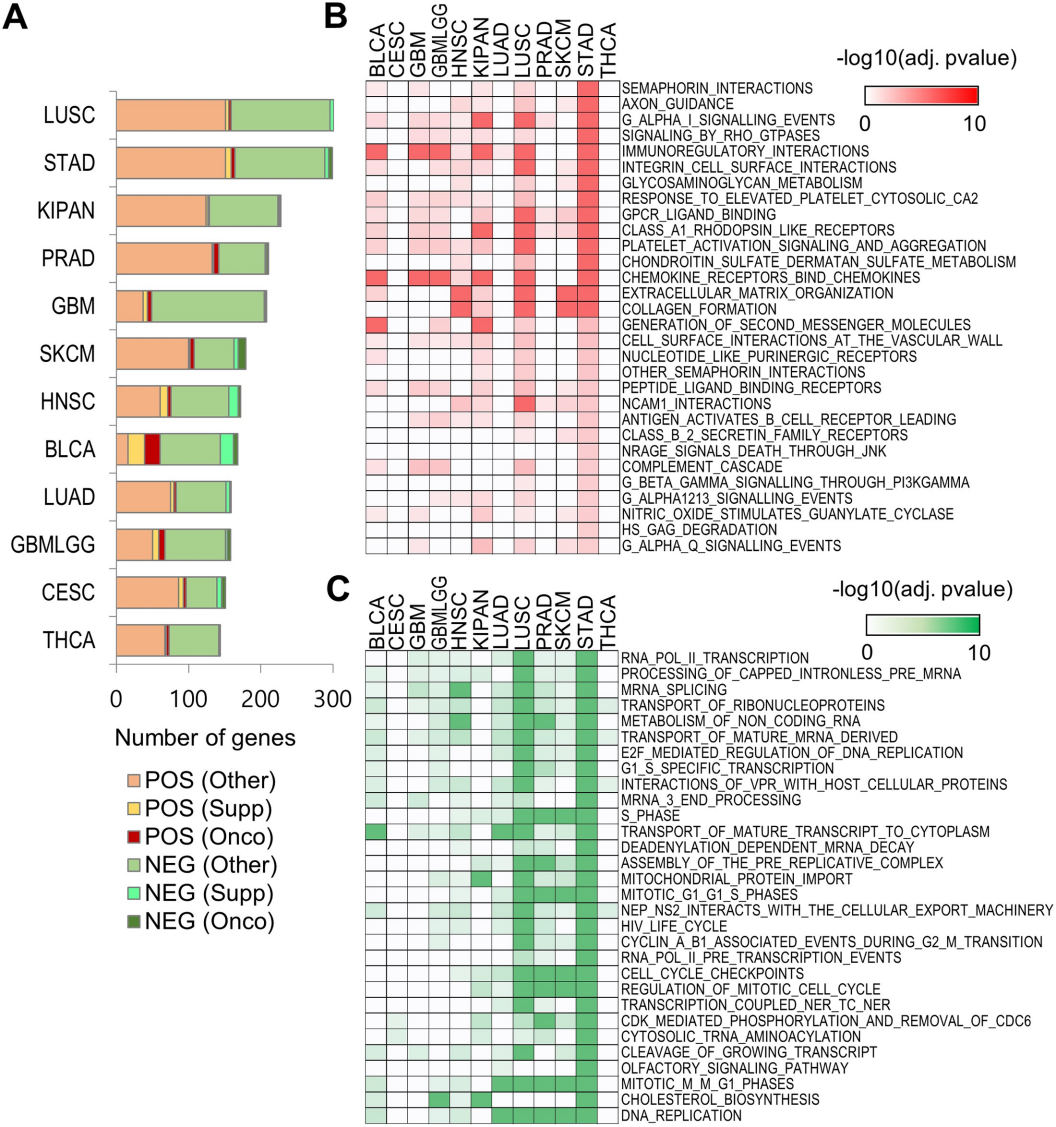
Association of RNA expression with ITH in 12 tumor types from TCGA. (A) Bar plot demonstrating comparison between the differential RNA gene expression in 12 tumor types from TCGA. BLCA: Bladder urothelial carcinoma; CESC: Cervical and endocervical cancers; GBM: Glioblastoma multiforme; GBMLGG: Glioma; HNSC: Head and neck squamous cell carcinoma; KIPAN: Pan-kidney cohort; LUAD: Lung adenocarcinoma; LUSC: Lung squamous cell carcinoma; PRAD: Prostate adenocarcinoma; SKCM: Skin cutaneous melanoma; STAD: Stomach adenocarcinoma; THCA: Thyroid carcinoma. POS: Positive; NEG: Negative; Onco: Oncogene; Supp: Suppress gene; Other: Other gene. (B) and (C) Heat map of functional categories of genes positively and negatively correlated with ITH, respectively.

Fundamentally, gene expression profiles have a functional importance for clonal evolution so that we have analyzed RNA expression profiles in 12 cancer types from the Broad GDAC Firehose (https://gdac.broadinstitute.org/). In our study, ITH was defined as the number of distinct tumor cell populations (subclones), measured by using EXPANDS and PyClone [10]. We performed the correlation analysis between clone number and gene expression profile in order to examine which cancer types are highly correlated to ITH. Overall expression profiles of many genes were significantly correlated (*p* < 0.01, Spearman’s rank correlation) in Lung squamous cell carcinmoma (LUSC) and Stomach adenocarcinoma (STAD) than other cancer types (Fig 1A). 303 (1.47% of total genes) and 299 genes (1.45%) were significant, respectively. Among them, several oncogenes and tumor suppressors were included (5 and 10 in LUSC, 10 and 13 in STAD). Next, we identified enriched functions associated with those genes. STAD showed the most prominent association with both positively and negatively correlated genes with ITH (Fig 1B and 1C). And there are more samples in STAD with clone than other cancer types. CESC and THCA did not have enriched function. Based on high correlation and available enough data for subclone numbers with ITH in STAD, we performed following analysis for genomic and transcriptomic features of stomach adenocarcinoma. Here, this cohort is Stomach adenocarcinoma (STAD) rather than Stomach and Esophageal carcinoma (STES) in the Broad GDAC.

### Characteristics of genomic and transcriptomic markers in ITH of stomach adenocarcinoma

**Fig 2:**
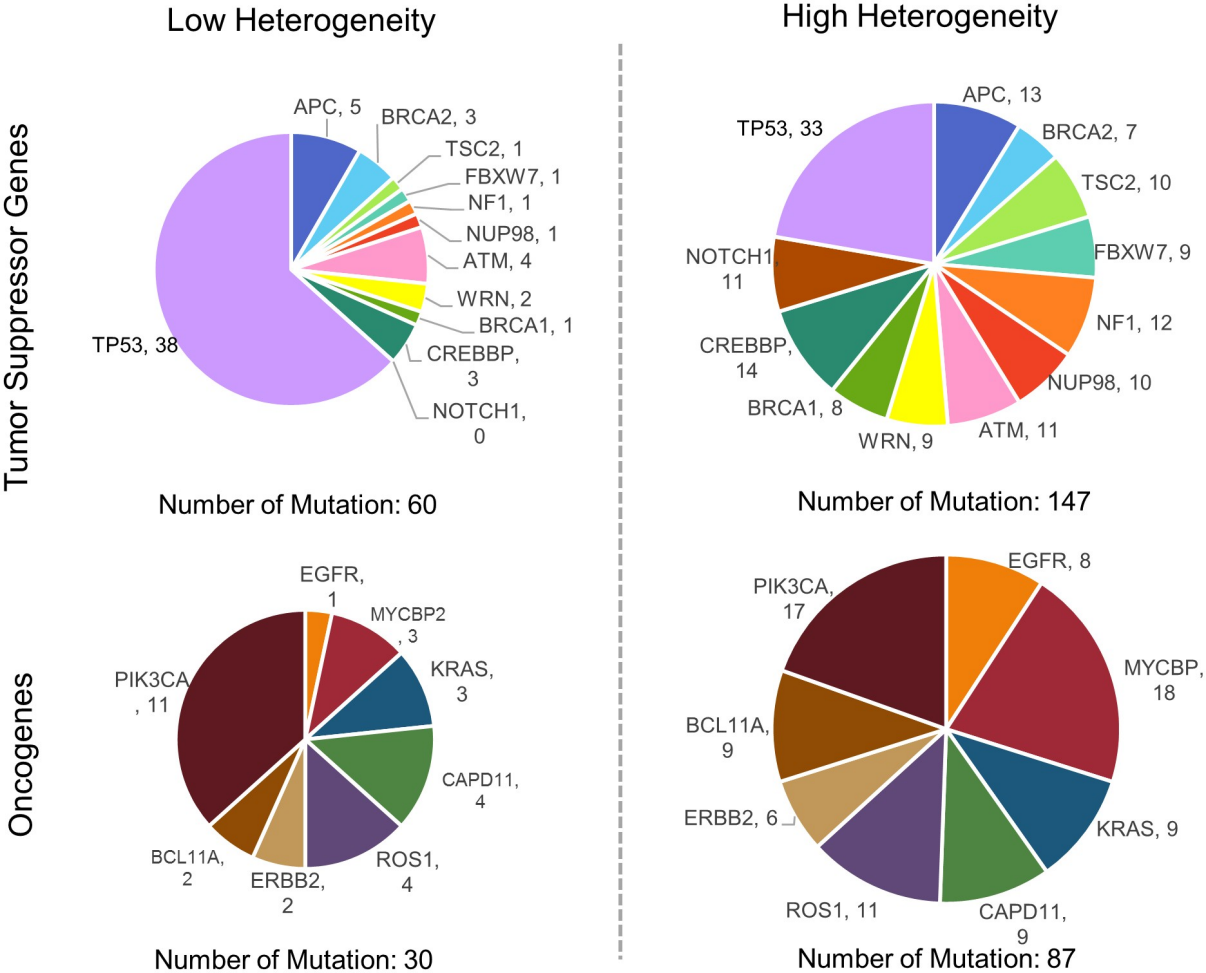
Pie charts representing the frequency of tumor suppressor genes and oncogenes between the low and high heterogeneity groups. Each gene name and its mutation count in a group are displayed.

A variety of approaches could be considered to determine the factors affecting the tumor heterogeneity of stomach adenocarcinoma. We examined the genomic and transcriptomic characteristics of 128 tumor samples with high heterogeneity and low heterogeneity. We assume that tumor suppressor genes and oncogenes are found frequently in tumors with high heterogeneity rather than in those with low heterogeneity. As expected, the distribution of both tumor-driver genes indicates that they were enriched in tumors with high heterogeneity (Fig 2). Among the 63 tumor suppressor genes, 12 genes had mutations and a total of 147 mutations were present in tumors with high heterogeneity. On the other hand, those genes harbored only 60 mutations in tumors with low heterogeneity. Most of the genes, except for *TP53*, had a higher mutation frequency in the high heterogeneity group. The frequency in oncogenes was also different between both the groups, showing more abundance (87 mutations) in the high heterogeneity group than in the low heterogeneity group (30 mutations). Among oncogenes, *MYCBP2* was the most prominent in the high heterogeneity group whereas *PIK3CA* was the most frequent in the low heterogeneity group (S1 Fig). In all the tumor stages, oncogene and tumor suppressor genes were prevalent in tumors with high heterogeneity (S2 Fig). We could confirm that there was genomic difference between both the groups in the tumor associated genes.

**Fig 3:**
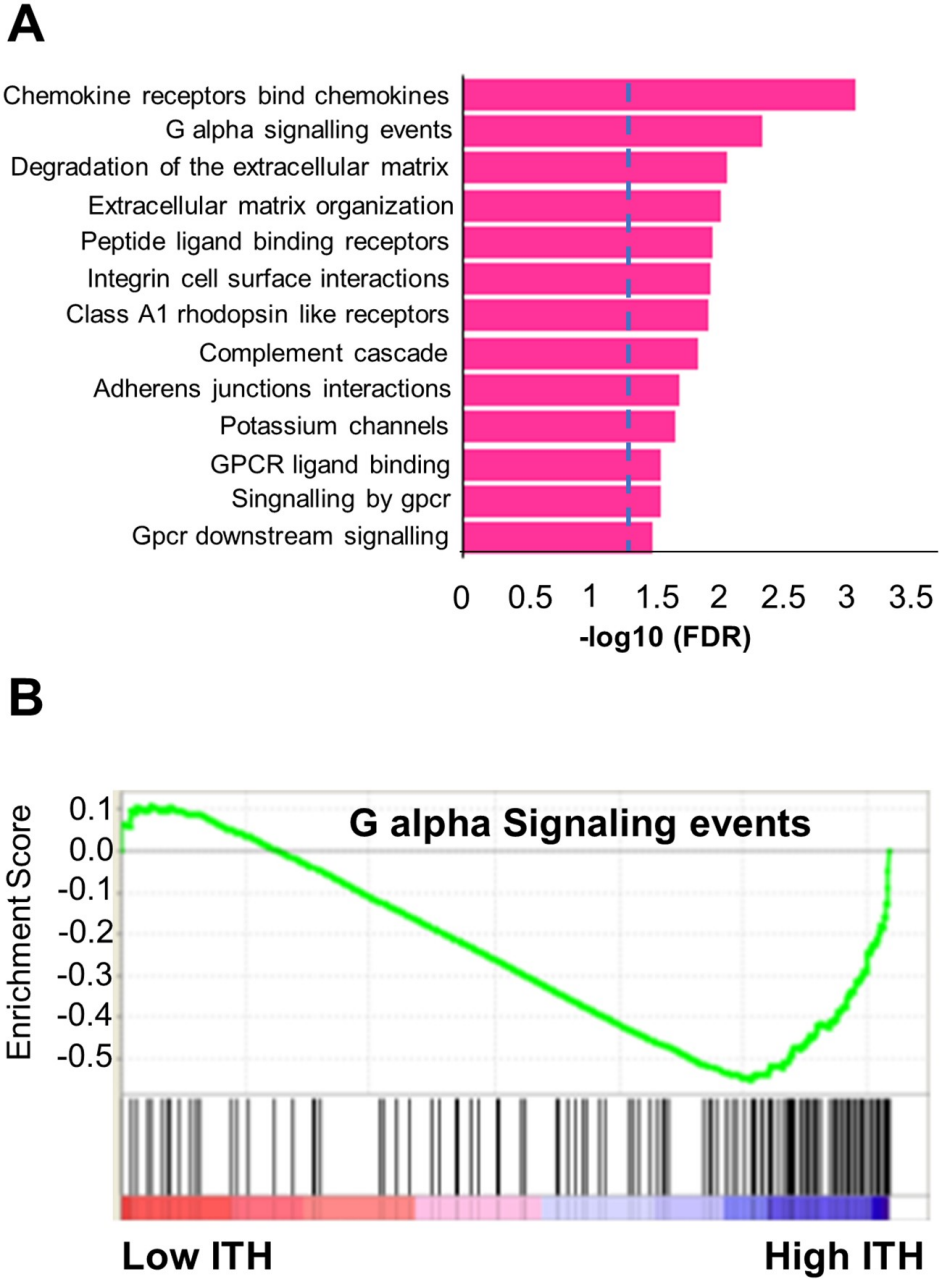
Functional differences between the low and high heterogeneity groups. (A) Enriched functions in genes showing expression difference between both the groups. (B) Enrichment of genes belonging to G alpha signaling events.

We also assume that the differences in the expression levels of many genes might cause functional differences associated with high intratumoral heterogeneity. Thus, we identified differentially expressed genes (DEGs) between the low and high heterogeneity groups using the R package, DEseq [11]. The *COL11A2* genes were highly expressed in the high heterogeneity group than in the low heterogeneity group and the *CLDN22* genes were expressed at low levels (> 2-fold change and adj p < 0.05). We were able to confirm the difference in the gene expression in both the high and low heterogeneity groups. Next, we identified enriched functions through GSEA (Gene Set Enrichment Analysis) (Fig 3A). We could find several pathways showing functional differences, including “G-protein alpha signaling”, “Extracellular matrix organization”, “Degradation of extracellular matrix”, “Integrin cell surface interactions”, “Signaling by GPCR” and “Adherence junctions interactions”. Among them, the G-protein alpha signaling pathway is mainly involved in signal transmission of cells [12] and low expression levels of genes in that pathway were associated with high ITH in our analysis (Fig 3B). Recent studies have shown that G protein alpha signaling affects the stomach adenocarcinoma growth through p53/p21 and MEK/ERK pathways [13].

### Performance comparison of classifiers

**Fig 4:**
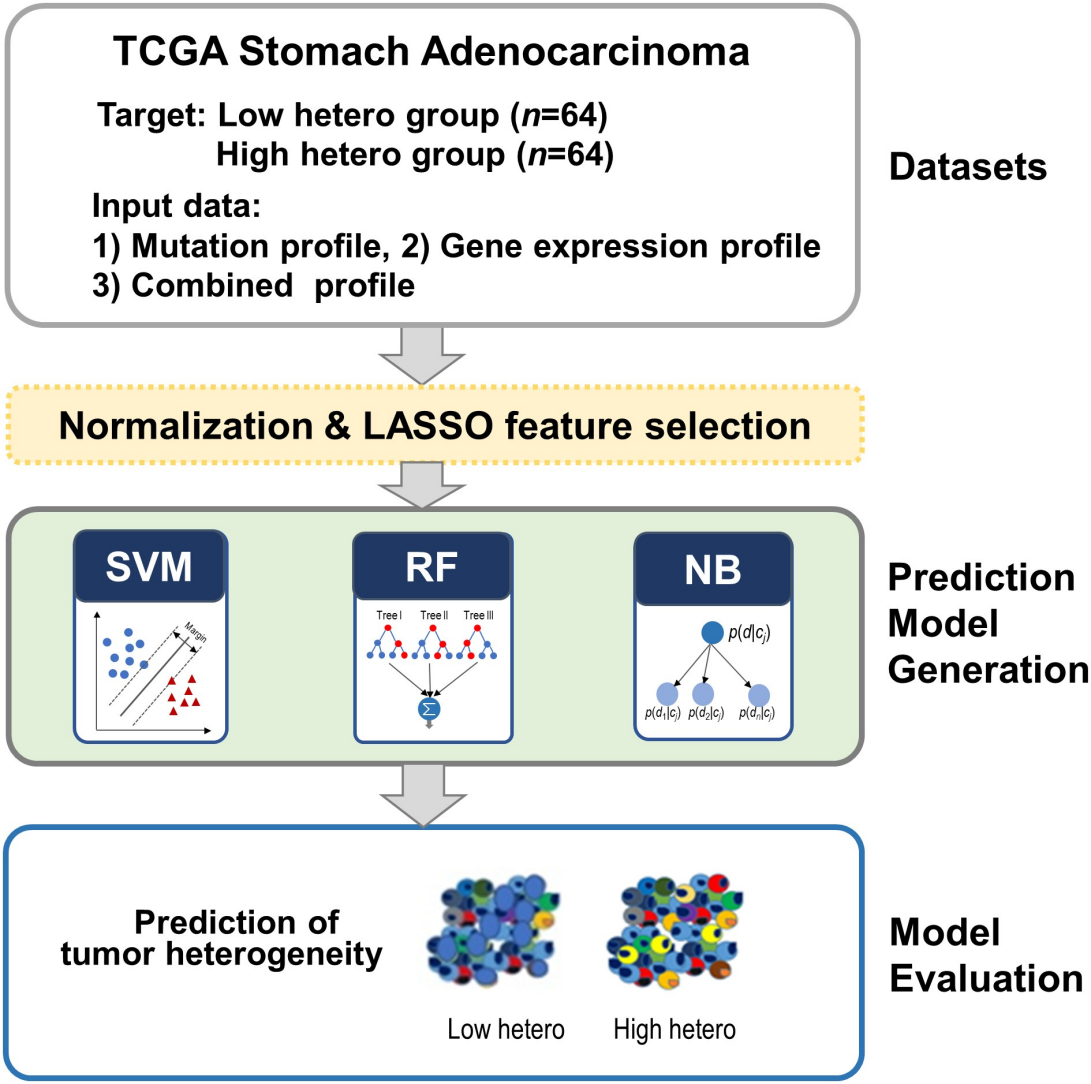
Schematic flow of tumor heterogeneity prediction. We performed machine learning in three steps: 1) preparation of mutation and RNA expression data set, 2) prediction model construction, and 3) evaluation of constructed model.

**Fig 5:**
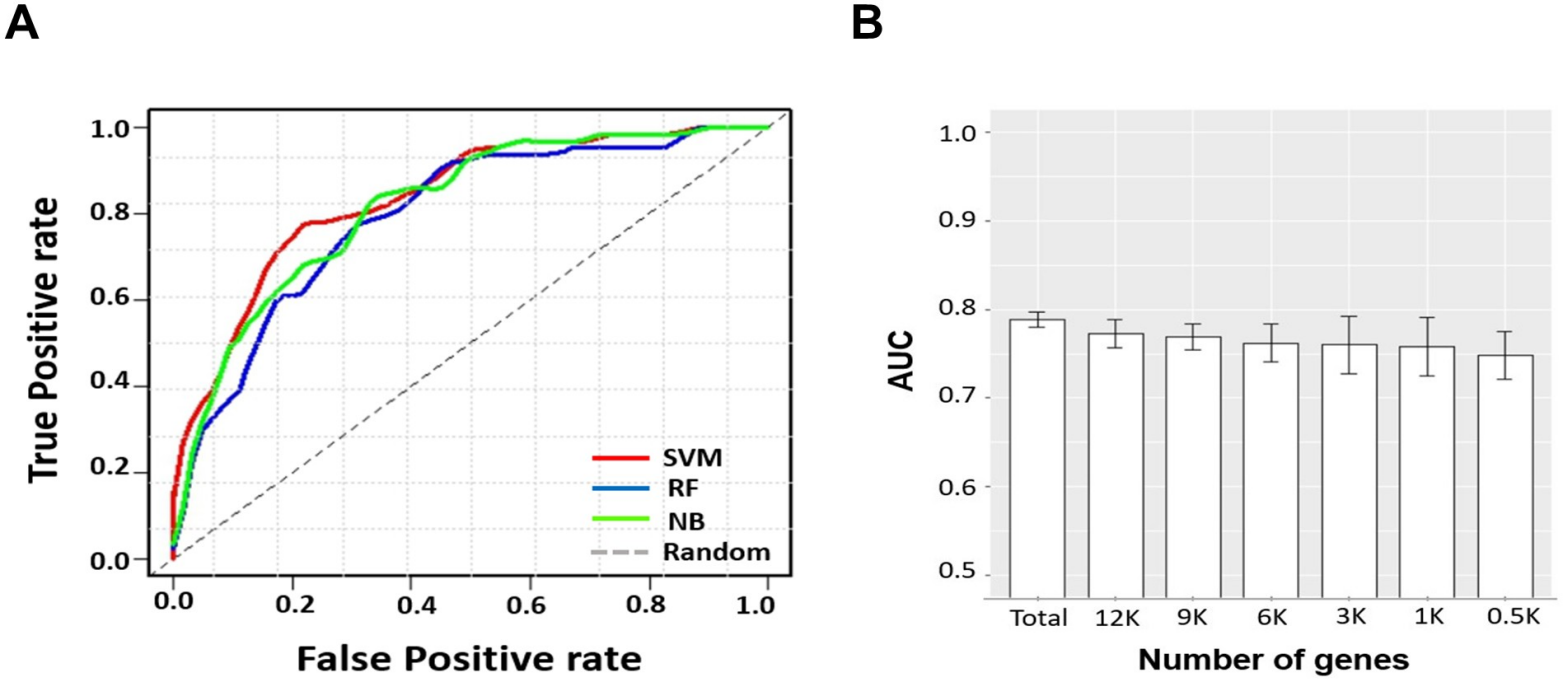
Performance comparison of classifiers. (A) Receiver operating characteristic curve of predictive performance on three classifiers. (B) Measurement of the performance with limited gene set in the RNA expression dataset with SVM.

We classified tumor samples into low and high heterogeneity groups by applying three prediction methods. Three types of datasets (mutation, RNA expression, and both the datasets combined) were tested (Fig 4). First, we compared the performance of the prediction methods according to the data types. The performance of each run was evaluated by 10-fold cross validation. The prediction accuracy is summarized in Table 1. Each value is the average of 10 runs. Overall, the performance was better when the two datasets were combined than when only one dataset was used. In terms of methods, the SVM outperformed the other methods for the two data types, exhibiting 71.02% in the mutation and 75.54% in the combined dataset. The SVM method achieved the highest area under the curve (AUC) (0.84) using the combined dataset (shown in Table 1 and Fig 5A). In the case of NB, the sensitivity in the combined dataset was the highest (0.90), but the specificity was the lowest (0.49). In conclusion, the best performance was when we applied SVM with the combined dataset.

**Table 1:** Comparison of the predictive performance according to the real datasets.

Data Type	Methods		
	Naïve Bayes	Support Vector	Random Forests
Mutation (Accuracy)	65.23%	71.02%	68.83%
RNA Expression (Accuracy)	71.33%	70.08%	69.84%
Combined Data (Accuracy)	69.38%	75.54%	72.87%
Combined Data (Sensitivity/Specificity)	0.90/0.49	0.73/0.78	0.75/0.69
Combined Data (AUC)	0.82	0.84	0.81

Next, we tested whether the prediction was stable when a limited subset of genes, out of the total set, was applied, implying loss of information. A subset of genes was randomly selected from the total number of genes. As expected, the performance with regard to the classification of heterogeneity was decreased with high variance with the decrease in the size of gene set when we tested with SVM (Fig 5B). This suggests that it is important to generate a prediction model with the most available features before the feature selection.

### Candidate genomic features for stomach adenocarcinoma with high heterogeneity

We were able to obtain a list of genes that contributed to the classification using the Lasso [14] feature selection method (shown in S1 Table). When we selected the top ranked genes, the *MKRN3* gene had the highest coefficient value in the mutation dataset and the *PDGFRA* gene had the highest value in the RNA expression dataset. Especially, the *PDGFRA* gene has been associated with tumor progression in stomach adenocarcinomas and has been associated with a poor prognosis [15]. The *PLEC* gene showed high difference in the mutation frequency between the low and high heterogeneity groups. The *PLEC* gene has been associated with binding of protein coding and actin binding. Also, *PLEC* is an important gene for cell pleomorphism and cytoskeleton disorganization [16].

### Performance test of predictive models with simulation data

We confirmed that the prediction model could distinguish the tumor heterogeneity when multiple tumor samples were randomly mixed. The simulation datasets were generated by using mutation profiles from the Cancer Cell Line Encyclopedia (CCLE) dataset and RNA-seq expression profiles from the CCLE and GTEx dataset, as described in the Methods section. A total of 150 mixed samples having diverse heterogeneity were used to evaluate the prediction models. As shown in Table 2, the performance was better in the RNA expression dataset than in the mutation dataset. Furthermore, the prediction models achieved the best performance in the combined dataset (showing an accuracy of 80.93% and AUC of 0.88 for SVM). This showed consistent results with the results obtained by using real datasets.

**Table 2:** Comparison of the predictive performance according to the data type on simulation datasets.

Data Type	Methods		
	Naïve Bayes	Support Vector	Random Forests
Mutation (Accuracy)	58.27%	61.40%	59.87%
RNA Expression (Accuracy)	69.80%	73.87%	68.87%
Combined Data (Accuracy)	73.47%	80.93%	76.80%
Combined Data (Sensitivity/Specificity)	0.65/0.81	0.81/0.81	0.77/0.76
Combined Data (AUC)	0.83	0.88	0.85

**Fig 6:**
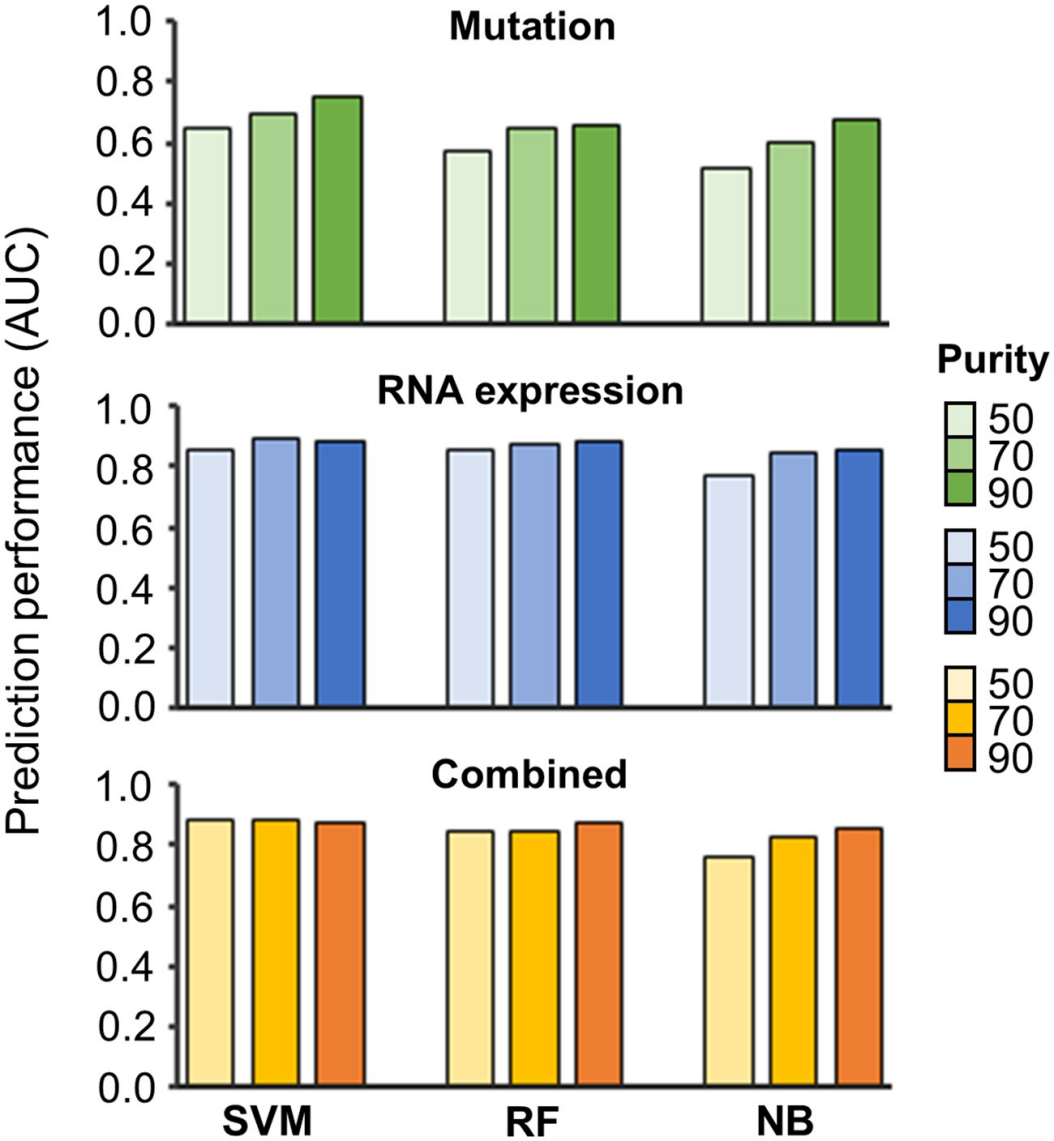
Measurement of the performance with limited gene set in the RNA expression dataset. The simulation shows the performance of each prediction method according to different tumor purity (50, 70 and 90).

Among the parameters related to the composition of tumor samples, the distribution of tumor purity might affect the prediction performance. We examined as to how the prediction performance changed with the average tumor purity (Fig 6). Overall, the prediction ability was affected by tumor purity when using the mutation profile, and tended to decline at the setting of low purity. Among the three algorithms, the performance of NB was affected for all the dataset types. However, the performance of SVM and RF showed a small change according to the setting of various tumor purities when using RNA expression and combined dataset. This indicates that those algorithms are more tolerant to diverse tumor purities when using the RNA expression dataset.

## Discussion

In this study, we suggest a novel framework to classify tumor samples according to their heterogeneity, representing the degree of different cell populations. We demonstrated the first prediction approach that could distinguish samples with heterogeneity using the mutation and RNA expression data, as well as a combination of both the data. Our prediction models showed reliable performance when using a real dataset and the results were also consistent with the simulation data. Our approach could be utilized to conveniently find tumor samples showing high heterogeneity as well as for easily finding the genomic and transcriptomic markers through feature selection.

Our approach has several advantages. First, for systematic analysis, tumor samples with high heterogeneity could be found conveniently. Second, our method could improve the performance of distinguishing heterogeneity through the addition of different types of data sets including mutation and transcriptomics datasets. Finally, the features associated with tumor heterogeneity can be automatically extracted.

We explicitly examined the characteristics of each group in detail by dividing the samples into groups with low and high heterogeneity. In the group with high tumor heterogeneity, mutations in the tumor suppressor genes and oncogenes were relatively more than in the group with low heterogeneity. Most mutations were detected in both high heterogeneity group and low heterogeneity group, with different percentage of mutations in *PIK3CA*, *KRAS* and *APC*. Those oncogenes were more found in high heterogeneity group than low group (*PIK3CA*: 26.6% in high-heterogeneity group and 17.2% in low-heterogeneity group; *KRAS*: 14.1% and 4.7%; *APC*: 20.3% and 7.8%). Previous study shows that mutational heterogeneity in *APC* and *KRAS* could cause polyclonality in early colorectal tumorigenesis [17]. Another study suggested that *PIK3CA*, *SMAD4* and *TP53* are most often associated with clonal divergence [18]. These driver genes may cause an increase of tumor heterogeneity via clonal expansion.

Also, the amount of RNA expression was significantly different between the high and low heterogeneity groups. Because there were apparently different genomic and transcriptomic alterations between both the groups, these important changes might contribute toward the high heterogeneity in tumor progression. Although the top-ranked mutated genes selected by feature selection are not well known in tumorigenesis, their occurrences were distinctly different between both the groups. The cause and effect of this association would prove invaluable for further studies.

There are different levels of ITH, e.g., phenotypic ITH such as heterogeneity in cell shape and morphology as well as non-genetic heterogeneity ITH such as heterogeneity in signaling [19] and epigenetic [20]. In this study, we focused on genetic aspect of ITH. However, considering different levels of ITH can help to understand the overall mechanism of tumor progress accurately and globally. In the future, it may be possible to conduct classification studies of ITH using these data.

In addition, there are the several issues on identifying ITH with single cell sequencing data. Recently they have been utilized for studying tumor heterogeneity [21–23]. The evaluation with single-cell RNA sequencing (scRNA-seq) data could be more reliable for studying ITH compared to that using bulk data. scRNA-seq analysis has an advantage that it can directly measure different tumor cell populations while analysis using the bulk sequencing analysis infers indirectly them. The profile of single cells can be deconvoluted with tools such as MuSiC [24] and CIBERSORT [25] to find out the cell type and the heterogeneity can be measured by grasping different molecular cell contents. This analysis will allow us to accurately evaluate tumor heterogeneity, as it provides more comprehensively the information of actual tumor microenvironment compared to analysis of bulk data or the mixture of cell lines.

As a future work, deep learning techniques will be applied [26]. The current machine learning approaches have a limitation with feature selection. To overcome this limitation and to improve the performance, we will try deep learning to classify ITH.

In summary, through the performance evaluation of the prediction models for ITH, we confirmed the plausibility of the proposed approach that could predict high tumor heterogeneity. We expect that this study will provide a novel insight for interpreting intratumoral heterogeneity so that it can be utilized for clinical implementation in diverse cancers.

## Methods

### Correlation analysis between ITH and gene expression

The Cancer Genome Atlas (TCGA) dataset (version 2016.01.28) for 12 cancer types was downloaded from Broad GDAC Firehose (http://firebrowse.org/). The full name and the abbreviation of these cancer types are as follows: bladder urothelial carcinoma (BLCA), cervical and endocervical cancers (CESC), glioblastoma multiforme (GBM), low grade glioma (GBMLGG), head and neck squamous cell carcinoma(HNSC), pan-kidney cohort (KIPAN), lung adenocarcinoma(LUAD), lung squamous cell carcinoma (LUSC), prostate adenocarcinoma (PRAD), skin cutaneous melanoma (SKCM), stomach adenocarcinoma (STAD), and thyroid carcinoma (THCA). To obtain information on ITH, we used the number of clones calculated by PyClone and EXPANDS from previous studies [10]. PyClone performs Bayesian clustering by grouping somatic mutations into putative subclones. It infers the cellular prevalence of somatic mutations by taking both variant allele frequencies and copy number alterations [7]. EXPANDS estimates the number of subclones based on hierarchical clustering analysis with cell-frequency probability distributions of somatic mutations [27]. The correlations between ITH (i.e. the number of clones) and gene expression were measured by Spearman’s correlation coefficient (PCC) and the significant genes (*p* < 0.01) were counted. In order to identify biological functions associated with ITH, gene set enrichment analysis (GSEA) (software.broadinstitute.org/gsea/) was performed on REACTOME pathway gene sets using pre-ranked gene list of PCC.

### ITH prediction using genomic and transcriptomic datasets

The overview of the present study is presented in Fig 4. We first collected genomic and transcriptomic datasets from stomach adenocarcinoma patients and combined them into a single dataset. The target values for prediction were determined by the number of clones. We employed a machine learning method to predict the heterogeneity in real data. Furthermore, to evaluate the accuracy of prediction, we applied our predictive model to hypothetically heterogeneous tumor datasets generated from mixed cell lines. Our prediction model achieved the best performance in the combined dataset and demonstrated the plausibility of predicting high heterogeneity in stomach adenocarcinoma.

### Evaluation data for ITH prediction

From TCGA stomach adenocarcinoma (STAD) cohort, we obtained the mutation profiles of 289 patients and RNA expression profiles of 450 patients. We selected 128 patients that had the same sample ID as that of data for mutation, gene expression, and clone number. To classify the tumors according to ITH in stomach adenocarcinoma, the label of tumors with high heterogeneity (number of clones ≥ 6) was set to 1 and the label of those with low heterogeneity (number of clones < 6) was set to 0, based on the presence of well-balanced labels for positive and negative sets (S3 Fig). We defined target labels, *y* = {*y*_1_, *y*_2_, …, *y*_*N*_}, *i* = 1, …, *N* and *y*_*i*_ ∈ {0, 1}, where *N* is the total number of patients. As input data for a model, three types of datasets were considered. The set of mutation was denoted by $M=\{m_{1},m_{2},\ldots ,m_{N_{m}}\}$, the set of expression was denoted by $E=\{e_{1},\;e_{2},\ldots ,e_{N_{e}}\}$, and the combined set of both was denoted by *EM* = *M* ∪ *E*, where *N*_*m*_, and *N*_*e*_ correspond to the total number of features for mutation and expression, respectively. The features with mutation frequency ≤ 5 were excluded and those with low expression (normalized read count < 20) over total samples were also excluded. When *X*(*M)* was the input dataset of mutation profile, it could be represented as a *N* × *N*_*m*_ matrix and other datasets could also be represented in the same way. Both the combined datasets have the features of *N*_*me*_ = 16,383, merged from the mutation dataset (*N*_*m*_ = 957) and the RNA expression dataset (*N*_*e*_ = 15,426).

### Machine learning methods

The feature selection was performed using the least absolute shrinkage and selection operator (LASSO) [14]. The LASSO regression has a function of variable selection that increases the prediction accuracy by reducing the regression coefficient [28], which is an advantage of ridge regression, and makes the regression coefficient values of irrelevant features easily zero at the same time. Therefore, Lasso regression is known as an analytical method that can provide high prediction accuracy as well as the interpretive power of variable selection. We used the R package ‘glmnet’ [29] to select the important features. We did feature selection using LASSO from the training data, generated a classification model, and evaluated the performance using a test set. When applying it to the combined data, the features were selected separately from the mutation and expression datasets, and the selected features were merged so as to use more than one type of dataset.

To evaluate the performance of tumor heterogeneity prediction from the selected features, we compared three classification algorithms of Support Vector Machine (SVM), Random Forest (RF), and Naïve Bayes [30–32]. The performance of classifiers was evaluated based on 10-fold cross validation and was compared in terms of Area Under Curve (AUC). It was performed in R (version 3.4.3) and the parameter setting is as follows:

1. We applied radial basis function (RBF) kernel to a SVM classifier using R package ‘e1071’ (https://cran.r-project.org/web/packages/e1071/). Two parameters, C and sigma, for the RBF kernel SVM were determined by cross-validation with grid search of C = {0.1, 10, 100} and sigma = {0.01, 0.25, 0.5, 1}.
2. We used R package ‘klaR’ (https://cran.r-project.org/web/packages/klaR) to apply the Naïve Bayes classifier.
3. We used the R package ‘randomForest’ [32] to collect decision trees from a random subset of the data with standard settings. We determined the best hyper-parameter setting for the number of trees and the minimum size of terminal node through cross-validation for the number of trees = {200, 500, 1000, 2000} and the size of nodes = {2, 3, 4, 5}.

### Generation of simulation data

To validate our predictive model using the simulation method, we downloaded mutation data of 38 STAD cell lines from CCLE (https://portals.broadinstitute.org/ccle) and mixed them to generate simulation datasets exhibiting tumor heterogeneity. With high or low heterogeneous tumors, a mutation profile of *i*-th sample, *X*_*i*_(*M*) was generated according to following procedure: 1) The number of clone, *N*_*c*_, was selected according to random number generation from Poisson distribution with the parameter *λ* = 6; 2) the tumor purity value, *P*_*t*_, was determined from normal distribution with mean value, ${\overset{-}{P}}_{t}=70$ and variance, σ^2^ = 100; 3) the size of *j*-th clone, *S*_*j*_, was determined by random number generation, where *S*_*j*_ ∈ [20, 100]; 4) the cell lines of *N*_*c*_ were randomly selected, the variant allele frequencies (VAFs) of each variant were mixed based on the proportion of clone size, and each mixed VAF was adjusted by purity; 5) finally, VAFs were converted to binary values by using *VAF*_*cutoff*_ = 0.03.

To generate mixed RNA expression datasets, we downloaded RNA expression data of tumor cell lines from CCLE (https://www.ebi.ac.uk/gxa/experiments/E-MTAB-2770/Results) as well as the RNA expression data of normal cell lines were downloaded from the GTEx Portal (https://www.gtexportal.org/home/datasets). We used 38 tumor cell lines and 2 normal cell lines. Each mixed expression profile was generated in the same way as described above except for procedure 4 and 5, which were modified as follows: 4) cell lines of *N*_*c*_ were randomly selected, expression value of each gene was mixed based on the proportion of clone size, and the mixed expression was adjusted by purity; 5) the expression values of normal samples were added to the mixed sample by considering the proportion of normal sample as 1 − (*P*_*t*_/100).

A combined dataset of mutation and RNA gene expression was generated by performing two types of procedures simultaneously with the same cells. The two procedures are shown in S4 and S5 Figs, respectively.

## Supporting information

S1 Fig Occurrence of mutation in tumor suppressor genes and oncogenes per tumor sample. (TIF)

S2 Fig Comparison between the occurrence of mutations in tumors with high and low heterogeneity. (TIF)

S3 Fig Number of positive and negative samples according to the cutoff value for the number of subclones. (TIF)

S4 Fig Procedure for generating simulation data of mutations. (TIF)

S5 Fig Procedure for generating simulation data of RNA gene expression. (TIF)

S1 Table Top ranked genes chosen by the LASSO feature selection from mutation and RNA gene expression data. (DOCX)
