# Supplementary figures and images for: Assessment of intratumoral heterogeneity with mutations and gene expression profiles
Source: PLoS One. 2019 Jul 16;14(7):e0219682. doi: 10.1371/journal.pone.0219682 (PMC6634409; doi:10.1371/journal.pone.0219682)

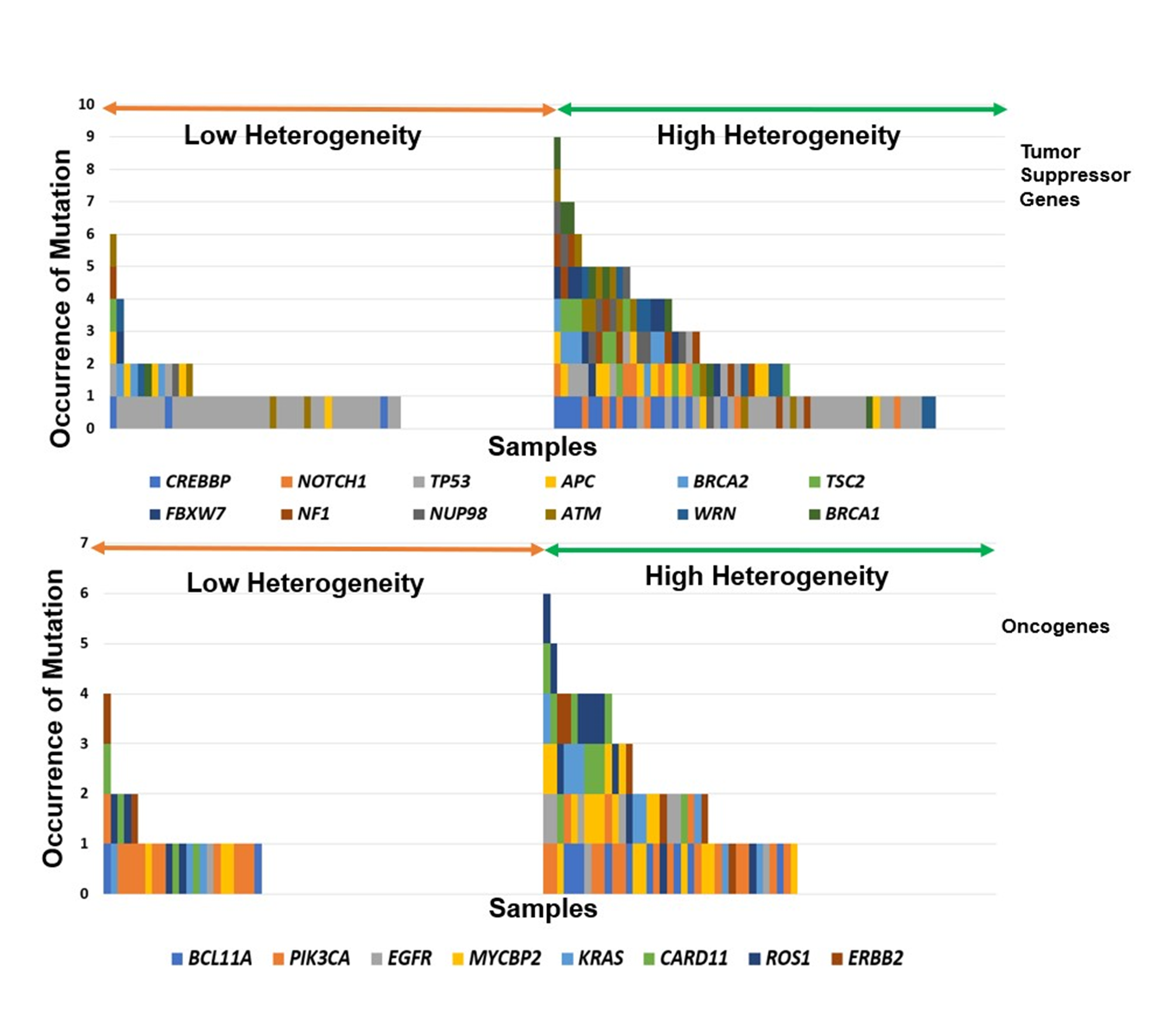

Supplement: S1 Fig — (TIF) [file pone.0219682.s001.tif]

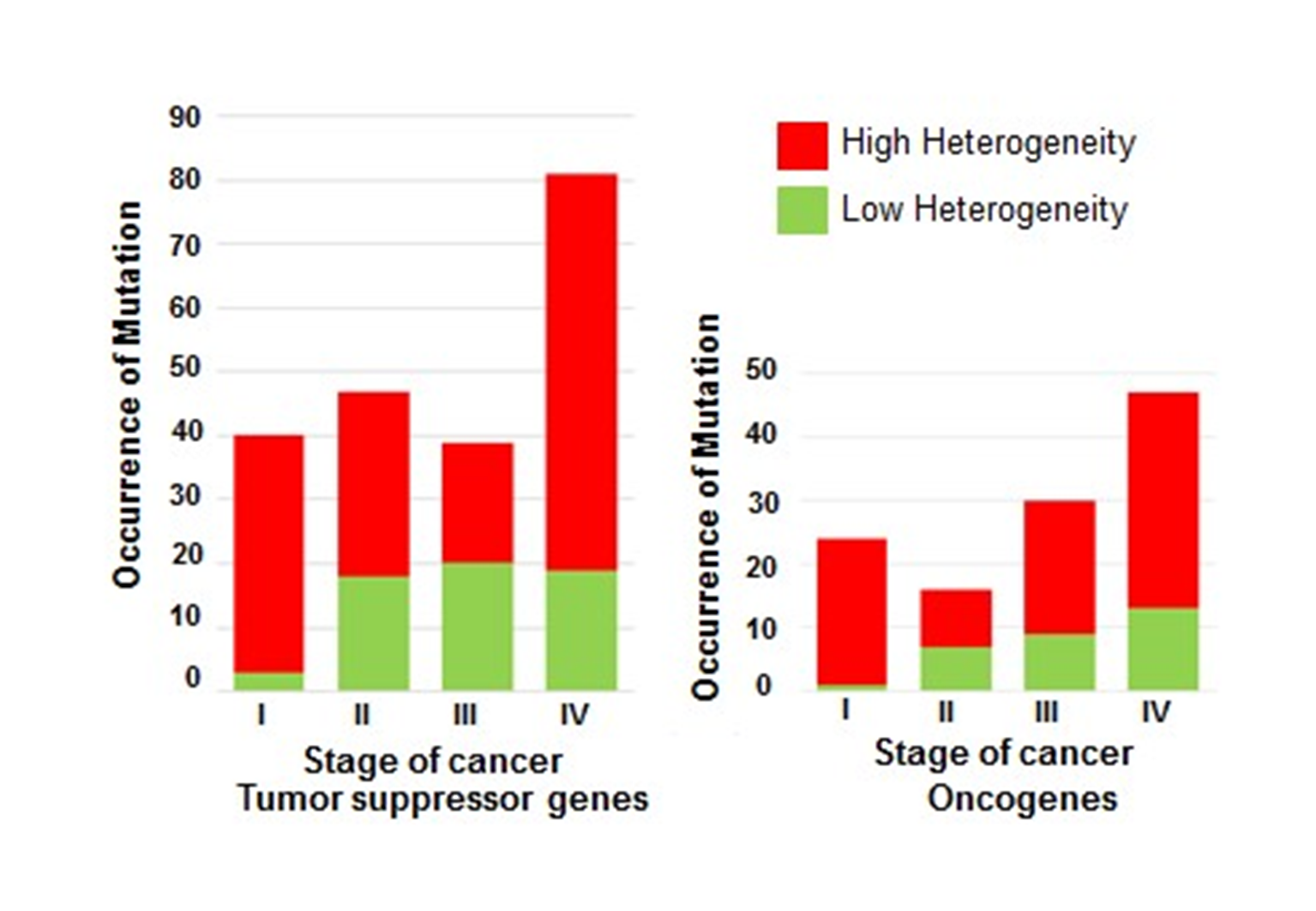

Supplement: S2 Fig — (TIF) [file pone.0219682.s002.tif]

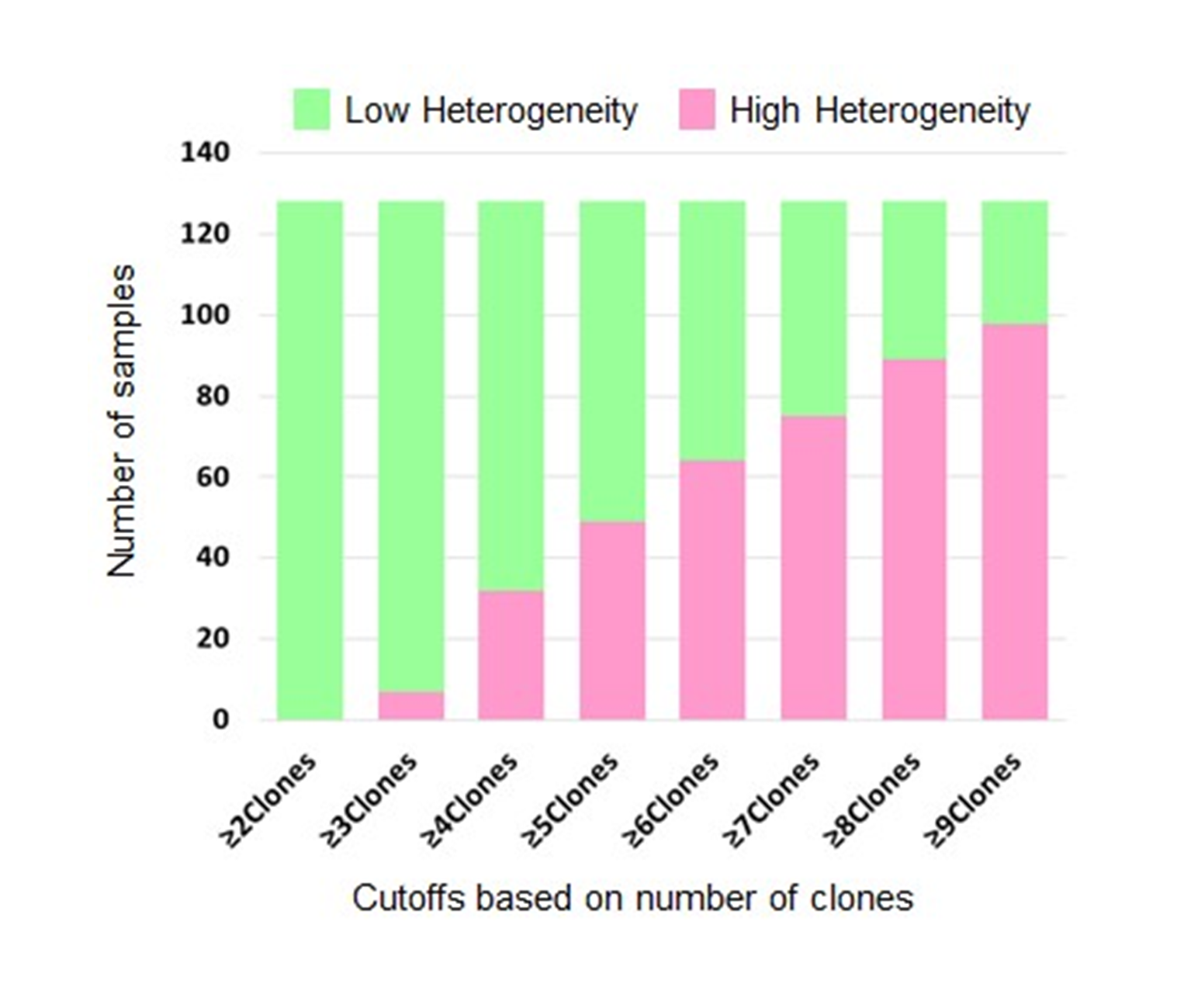

Supplement: S3 Fig — (TIF) [file pone.0219682.s003.tif]

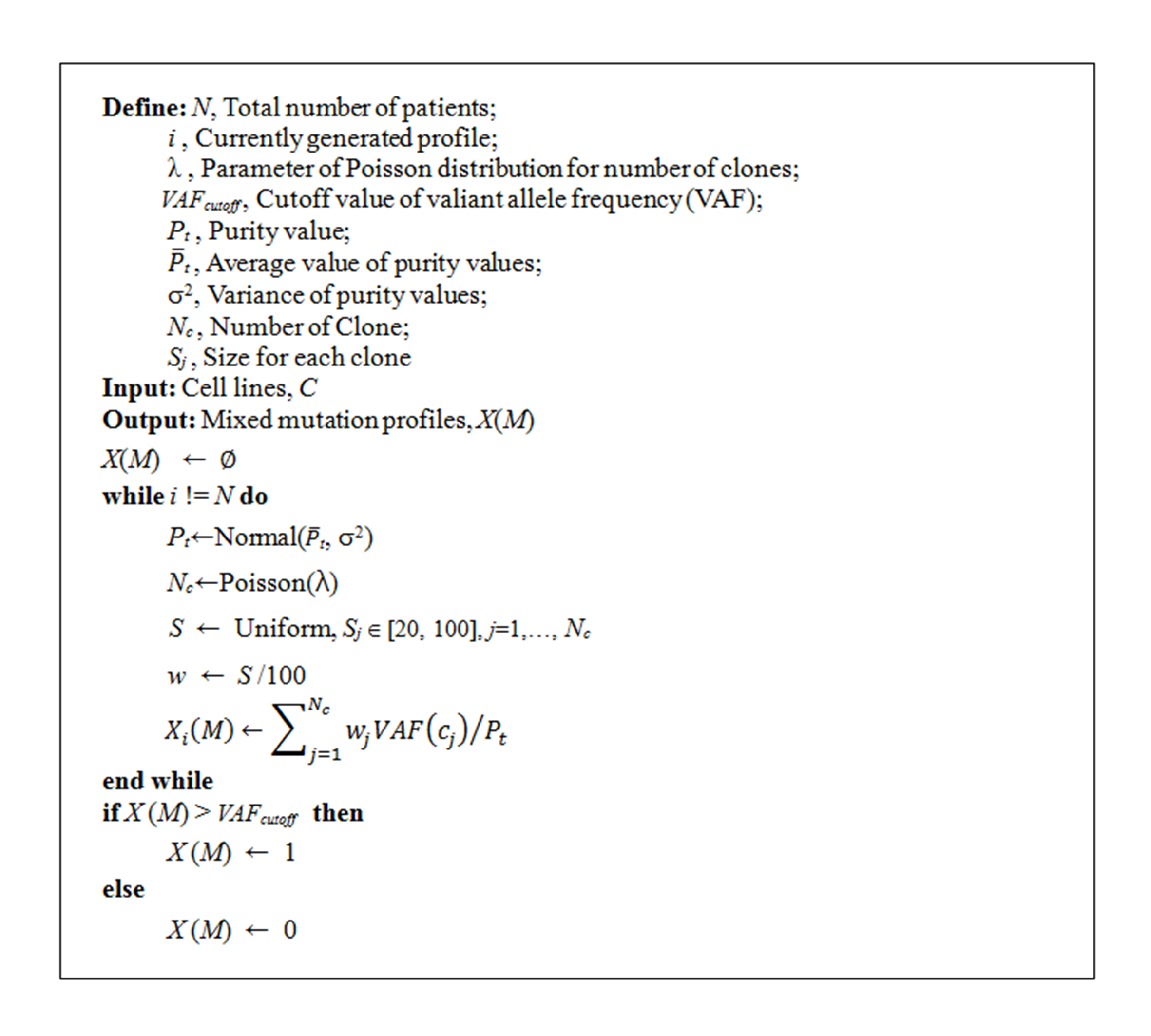

Supplement: S4 Fig — (TIF) [file pone.0219682.s004.tif]

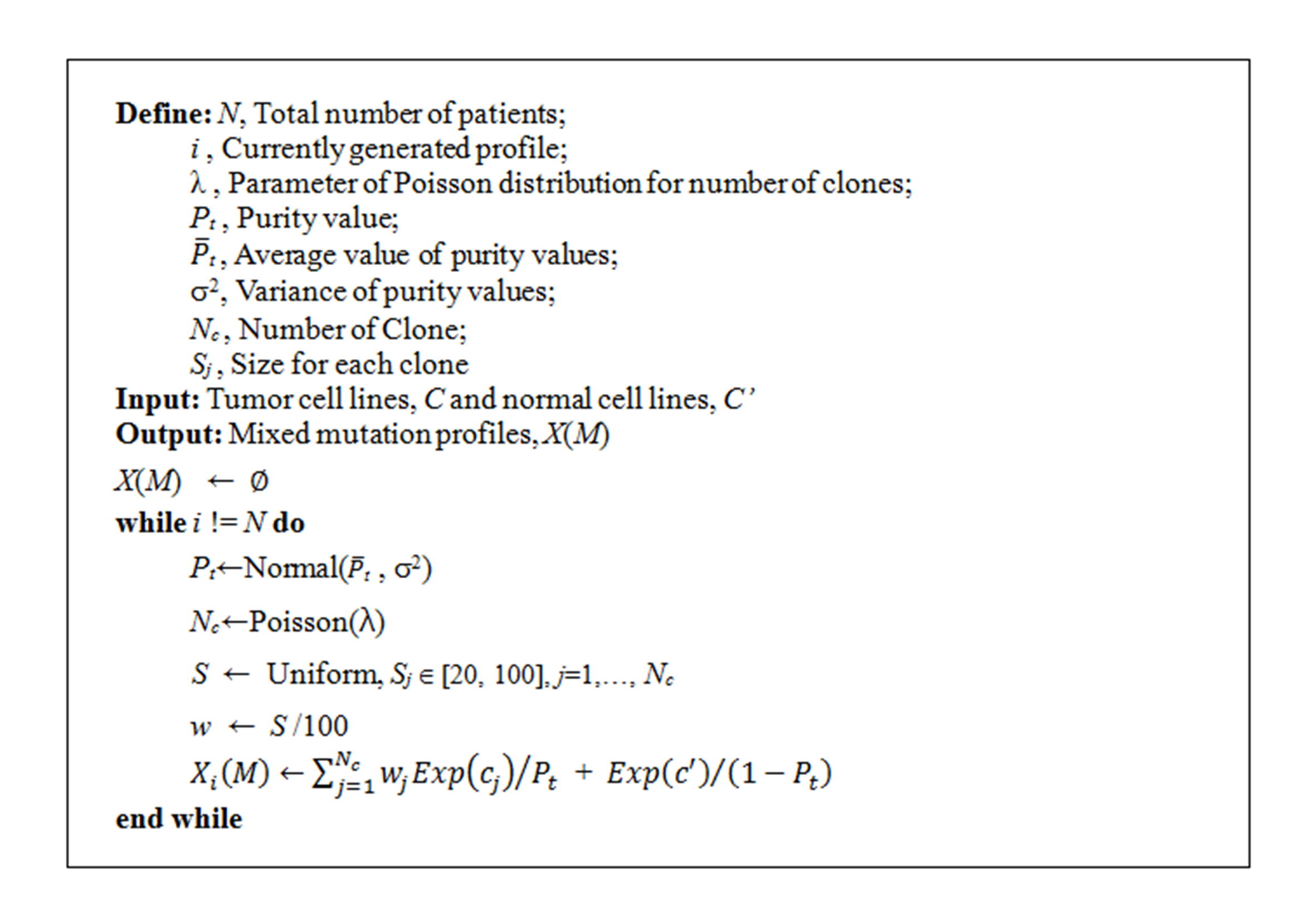

Supplement: S5 Fig — (TIF) [file pone.0219682.s005.tif]
