# Supplementary material for: Assessment of intratumoral heterogeneity with mutations and gene expression profiles
Source: PLoS One. 2019 Jul 16;14(7):e0219682. doi: 10.1371/journal.pone.0219682 (PMC6634409; doi:10.1371/journal.pone.0219682)
Supplement: S1 Table — (DOCX) [file pone.0219682.s006.docx]

**Table S1.** Top ranked genes chosen by the LASSO feature selection from mutation and RNA gene expression data.

| Mutation | Low  Hetero^*^ | High  Hetero^*^ | Coefficient | RNA Expression | Low  Hetero^†^ | High  Hetero^†^ | Coefficient |
| --- | --- | --- | --- | --- | --- | --- | --- |
| *MKRN3* | 0 | 14 | 0.211 | *PDGFRA* | 2286.88 | 1041.33 | 0.061 |
| *ZNF521* | 9 | 8 | 0.151 | *CAPN11* | 20.25 | 4.66 | 0.054 |
| *BZRAP1* | 3 | 14 | 0.134 | *INVS* | 308.39 | 269.73 | 0.052 |
| *UNC13C* | 0 | 18 | 0.126 | *RPH3A* | 3.51 | 9.00 | 0.051 |
| *DSCAM* | 2 | 14 | 0.119 | *CD40LG* | 40.14 | 25.12 | 0.050 |
| *FHOD3* | 2 | 12 | 0.117 | *C2orf74* | 107.38 | 63.83 | 0.046 |
| *PPP1R3A* | 1 | 9 | 0.105 | *MMACHC* | 176.42 | 238.23 | 0.046 |
| *ZNF831* | 0 | 15 | 0.105 | *HEATR6* | 362.67 | 317.21 | 0.045 |
| *PLEC* | 4 | 26 | 0.101 | *CBWD1* | 526.43 | 530.05 | 0.044 |
| *ATP10D* | 1 | 11 | 0.100 | *GUSBP1* | 77.39 | 101.10 | 0.044 |
| *CNTNAP5* | 2 | 15 | 0.091 | *PTOV1* | 1785.39 | 2173.50 | 0.044 |
| *SCN5A* | 1 | 16 | 0.085 | *ARHGAP28* | 105.39 | 55.30 | 0.044 |
| *TRPM1* | 1 | 14 | 0.085 | *RGS7* | 16.33 | 6.27 | 0.043 |
